# Development of NADES–Annatto Seed Extract for Enhancing 3D Printed Food Designed for Dysphagia Patients

**DOI:** 10.3390/foods14091604

**Published:** 2025-05-01

**Authors:** Sara Kierulff Balabram, Larissa Tessaro, Maria Eduarda de Almeida Astolfo, Pedro Augusto Invernizzi Sponchiado, Stanislau Bogusz Junior, Bianca C. Maniglia

**Affiliations:** São Carlos Institute of Chemistry, University of Sao Paulo, Av Trabalhador São Carlense, 400, São Carlos 13566-590, SP, Brazil; sarakbalabram@usp.br (S.K.B.); larissa.tessaro@usp.br (L.T.); maria.e.astolfo@usp.br (M.E.d.A.A.); pedrosponchiado@usp.br (P.A.I.S.); stanislau@iqsc.usp.br (S.B.J.)

**Keywords:** natural deep eutectic solvent (NADES), 3D food printing, annatto seed extract, dysphagia-friendly foods, texture profile analysis (TPA), hydrogel-based food, bioactive compounds, food rheology, functional food design, IDDSI framework

## Abstract

This study develops a 3D printed food designed for dysphagia patients, incorporating a natural deep eutectic solvent (NADES)–annatto seed extract. The objective was to enhance textural properties and bioactive retention in food matrices tailored for individuals with swallowing difficulties. NADES extraction was compared to ethanol, with the extracts incorporated into gelatin and starch hydrogels. Gelatin, a widely used biopolymer, improved mechanical properties and printability, ensuring a cohesive and structured matrix for 3D printing. Textural analysis showed that starch-based 3D printed hydrogels exhibited lower hardness, adhesiveness, and gumminess compared to molded samples, making them more suitable for dysphagia-friendly diets than gelatin-based formulations. The IDDSI fork test confirmed that selected 3D printed samples met essential texture requirements for safe consumption by dysphagia patients. The combination of NADES-extracted bioactive compounds and 3D printing enabled the development of functional foods with optimized texture and nutritional properties. Additionally, gelatin played a key role in enhancing elasticity and structural integrity in printed samples, reinforcing its potential for food texture modification. This study presents an innovative approach to dysphagia-friendly food formulation, integrating green extraction methods with advanced food processing technologies, paving the way for safer, nutritionally enhanced, and customizable functional foods for individuals with swallowing disorders.

## 1. Introduction

Dysphagia, or difficulty chewing/swallowing, is a prevalent condition, particularly among the elderly and individuals with neurological impairments, which can significantly impact the nutritional and physiological health of these people [1,2]. This occurs because dysphagic patients experience loss of teeth, weakness in the muscles responsible for chewing and the tongue, or impairment of other physiological functions, which lead to an abnormal delay in swallowing [3]. Because of this, food for dysphagic patients tends to be pasty, in the form of purees and viscous liquids, making them visually unattractive and impacting the appetite [4]. Developing foods for dysphagic people that are not only easy to swallow but also nutritionally balanced, visually attractive, and capable of maintaining their texture during consumption is a challenge [5]. Three-dimensional food printing has emerged as a promising technology, enabling precise control of food textures and shapes to meet the individual needs of this group of people [3,4]. This cutting-edge technology deposits ink layer by layer [6] and can create stable 3D printed foods with desirable textures and diverse shapes [3].

Viscoelastic ingredients such as dough, paste, and other gel-forming substances can be used as ink for 3D printing of food by extrusion [7]. The use of biopolymers such as gelatin and starch in the formulation of edible inks to produce these printed foods stands out. Both gelatin and starch are widely used in the food industry due to their ability to form hydrogels and modify the texture of food [4,8], in addition to being extracted from natural and renewable sources. Gelatin is a protein of animal origin, extracted by acid or basic hydrolysis of collagen from bone and connective tissues of some animals, such as bovine and porcine [9]. Because it is a hydrocolloid, gelatin is commonly used as a gelling agent in gums and other foods [10]. Starch is a polysaccharide made up of amylose and amylopectin, being one of the most important carbohydrates in the human diet, and is generally extracted from vegetable sources such as potatoes, rice, and cassava [6,11]. For food products, starch is gelatinized during cooking and used as a gelling agent [7].

It has been reported that both gelatin and starch have rheological properties necessary to be used as ink in extrusion 3D printing [7,12,13,14,15], which can be improved with the addition of other ingredients and/or chemical modifications [6,7,8,14]. Ink for 3D printing must have good fluidity for extrusion and viscoelasticity to form continuous lines. It needs to have adequate yield stress for shear during extrusion and low viscosity to facilitate the process, quickly acquiring structural strength after extrusion [13]. Furthermore, 3D printed foods must have some nutritional value, which does not only happen with formulations based on gelatin and starch. One strategy to make these foods more nutritious is to add substances rich in bioactive compounds [15], such as carotenoids and phenolic compounds, to these formulations, which will benefit human health. A plant source rich in carotenoids and other bioactive compounds, which can be used for this purpose, is the annatto seed, widely used to produce food colorings [16].

Annatto (*Bixa orellana* L.), from the Bixaceae family, is a tree native to South America but found in several regions of the world with a tropical climate, whose fruit seeds are widely used to produce natural food colorings [17]. Annatto fruits are pods that contain 10 to 20 seeds, with the seed shells being rich sources of carotenoids (over 50%) and other bioactive compounds [16]. Carotenoids are pigmented, fat-soluble compounds, and bixin (C_25_H_30_O_4_) is the main carotenoid present in annatto seeds (around 80%), responsible for the intense red-orange color [17,18]. Other carotenoids can be found in annatto seeds, such as norbixin, isobixin, and β-carotene, as well as some terpenoids and phenolic compounds [17]. The interest in these carotenoids and other bioactive compounds in food applications is attributed to the biological activities of these compounds (e.g., antioxidant, anti-inflammatory, and antimicrobial), which have been related to reducing the risks of various chronic-degenerative and inflammatory diseases [19,20]. In particular, bixin is known to be a very efficient agent in reducing oxidation reactions [19].

Traditional methods for extracting bixin and other bioactive compounds from annatto seeds use organic solvents, such as methanol, ethanol, acetonitrile, chloroform, and acetone, in addition to supercritical fluids [18,21,22,23]. Despite good extraction yields, these solvents cannot be applied to food products due to toxicity and adverse health effects. In this context, natural deep eutectic solvents (NADESs) can be a good green alternative for extracting bioactive compounds from annatto seeds. NADESs are formed by mixing two or more hydrogen bond acceptors (HBAs) and hydrogen bond donors (HBDs), which, at the eutectic point, change from the solid state to the liquid state at temperatures lower than the melting point of each isolated component [24]. They can be produced with secondary metabolites of natural origin, such as urea, carboxylic acid, sugars, amino acids, choline chloride, and polyols [25,26], which are non-toxic and edible. To the best of our knowledge, NADESs have not yet been used to extract bioactive compounds from annatto seeds, which represents the originality of this study.

In this study, we hypothesized that the application of a NADES–annatto seed extract in inks based on bovine gelatin and potato starch, in addition to bringing nutritional value, could improve rheological and printability properties, aiming to produce foods suitable for people with dysphagia. Therefore, our study focused on (i) producing an annatto seed extract using NADESs based on choline chloride and lactic acid and characterizing it in terms of its physical and bioactive properties; (ii) adding the NADES–annatto seed extract to hydrogels based on bovine gelatin and potato starch of the same concentrations to be used as inks for 3D printing by extrusion and evaluating the effect on rheological, mechanical, and printability properties; and (iii) producing 3D printed gummies based on these inks and evaluate the effect of adding the NADES–annatto seed extract on the texture properties and suitability for people with dysphagia compared to the properties of molded gummies with the same formulations.

## 2. Materials and Methods

### 2.1. Material

Annatto seeds were purchased at a local market (São Carlos, SP, Brazil). Bovine gelatin (type B, bloom 250) and potato starch (moisture content ≈ 16%) were donated by Gelnex by Darling Ingredients (Itá, SC, Brazil) and Zona Cerealista (São Paulo, SP, Brazil), respectively. For NADES preparation, choline chloride and lactic acid were supplied by Sigma-Aldrich (St. Louis, MO, USA). For antioxidant analyzes and quantifications, the following chemical reagents were used: β-carotene (CAS: 7235-40-7), Iron (III) chloride hexahydrate, 2,4,6-Tris(2-pyridyl)-s-triazine (TPTZ), (±)-6-Hydroxy-2,5,7,8-tetramethylchromane-2-carboxylic acid (Trolox), 2,2′-Azino-bis(3-ethylbenzothiazoline-6-sulfonic acid (ABTS), Folin–Ciocalteu phenol reagent, Nile Red (Sigma-Aldrich, St. Louis, MO, USA), anhydrous gallic acid and absolute ethyl alcohol P.A. (Êxodo Científica, Sumaré, SP, Brazil), butylated hydroxytoluene (Neon, Suzano, SP, Brazil), and hexane P.A. (Synth^®^, Diadema, SP, Brazil).

### 2.2. Preparation and Characterization of NADES

#### 2.2.1. NADES Preparation

NADES was produced by mixing choline chloride and lactic acid (CC:LA) at a 1:1 ratio (80% *w*/*w*) and distilled water (20% *w*/*w*) using an ultrasonic bath a potency of 100 W and a frequency of 40 kHz (Tabletop 404 digital, Delta Ultrassons, Diadema, SP, Brazil) at 50 °C for 45 min, according to Bertolo et al. [25], with slight modifications. The produced CC:LA was stored at 25 °C for characterization. A 60% (*v*/*v*) ethanolic solution (etOH-60%) was used as a control solvent for the extraction of active compounds from annatto seeds for comparison with CC:LA [26]. This solution was also kept at 25 °C for characterization.

#### 2.2.2. pH and Density

The pH of CC:LA and etOH-60% was determined at 25 °C using a portable pHmeter (K39-220, Kasvi, Pinhais, SP, Brazil), while the density was obtained by pycnometry at 25 °C, weighing the mass of CC:LA and etOH-60% in 5 mL pycnometers.

#### 2.2.3. Viscosity

The viscosity of CC:LA was obtained using a controlled stress rheometer (AR-1000N, TA Instruments, New Castle, DE, USA) with a Peltier system for temperature control. Steady-state flow measurements were performed using a cone-plate geometry with a cone angle of 2°, 40 mm diameter, gap of 55 μm, fixed shear rate of 1 s^−1^, and temperature ranging from 20 to 50 °C [26].

#### 2.2.4. Polarity

The polarity of CC:LA and etOH-60% was determined via the solvatochromic method using a UV-Vis spectrophotometer (UV-M51, BEL^®^ Engineering, Monza, MB, Italy), according to the method described by Fernandes et al. [27], with slight modifications [25]. An ethanolic solution of Nile Red dye 1 mg mL^−1^ was added to CC-LA and etOH-60% (1:200 dye–solution), and a wavelength scan was performed from 800 to 200 nm. The maximum absorption wavelength (λ_max_) of each solution was used to calculate the polarity parameter (E_NR_) in triplicate (Equation (1)) [28].

(1) $$E_{NR}\;\left({\mathrm{kcal}\;\mathrm{mol}}^{-1}\right)=\frac{28591.44}{\lambda_{max}}$$

where the E_NR_ (kcal mol^−1^) is the transition energy.

### 2.3. Production and Characterization of NADES–Annatto Seed Extract

#### 2.3.1. Production of NADES–Annatto Seed Extract

NADES–annatto seed extract (NE) was produced by ultrasound-assisted extraction, by mixing 1 g of annatto seeds with 15 mL of CC:LA using an ultrasound bath at 50 °C for 45 min [23,25]. After the extraction period, the sample was filtered through a paper filter (weight 80 g m^−2^), and the NE obtained was stored in the absence of light and analyzed immediately. As a control, an ethanolic annatto seed extract (EE) was produced using the same conditions, replacing the CC:LA with an etOH-60%.

#### 2.3.2. pH, Viscosity, and Color

The pH of NE and EE was measured using a portable pHmeter, and the viscosity of NE was obtained according to the methodology described in Section 2.2.3 using a controlled stress rheometer.

The color parameters (L*, a*, and b*) were obtained using a colorimeter (Delta Vista d.8, Delta Color, São Leopoldo, RS, Brazil) in reflectance mode (CIELab scale, illuminant D65, angle 10°, measuring opening 16 mm). Anglo Hue (h*) and Chroma (C*) were calculated by the equipment, according to Equations (2) and (3), respectively.

(2) $$h^{*}=\arctan\left(\frac{b^{*}}{a^{*}}\right)$$

(3) $$C^{*}=\sqrt{{\left(a^{*}\right)}^{2}+{\left(b^{*}\right)}^{2}}$$

#### 2.3.3. Bixin Quantification by High-Performance Liquid Chromatography (HPLC)

The bixin content in NE and EE was analyzed using a high-performance liquid chromatography (HPLC) system (LC20AD, Shimadzu, Kyoto, Japan) equipped with a photodiode array (PDA) detector (SPD-6AV, Shimadzu, Kyoto, Japan) and an Agilent Eclipse XDB-C18 column (4.6 mm × 250 mm, 5 μm particle size) (Agilent, Santa Clara, CA, USA). Before analysis, the samples were diluted in methanol and filtered through a 0.45 μm membrane (Millex, Darmstadt, Germany). The chromatographic method was adapted from Chisté et al. [19] and used a mobile phase consisting of water–phosphoric acid with 0.1% EDTA 4% (pH 3.0) (solvent A) and methanol–phosphoric acid with 0.1% EDTA 4% (pH 3.0) (solvent B). The elution was carried out at a flow rate of 1.0 mL min^−1^ at 40 °C for 10 min. UV–Vis spectra were recorded in the range of 200–600 nm, with bixin detection at 460 nm. The bixin content in the samples was identified by comparing UV–Vis spectra and retention times with external standards. Quantification was performed using six-point calibration curves from standard solutions, prepared in triplicate, with concentrations ranging from 0.5 to 5.0 μg mL^−1^.

#### 2.3.4. Total Carotenoids and Norbixin Content

The total carotenoid content of NE and EE was determined as described by Lüdtke et al. [29] with slight modifications. Briefly, 2.5 mL of NE or EE was vortexed with 5 mL of 0.01% butylated hydroxytoluene in acetone. Then, 5 mL of hexane was added and vortexed for 10 s. Distilled water was added to make the final volume of 25 mL, and the samples were centrifuged at 10,000 rpm and 4 °C for 5 min by a high-speed refrigerated centrifuge (CR22GIII, Hitachi, Tokyo, Brazil). The hexane supernatant was analyzed by a UV-Vis spectrophotometer at 450 nm. The results were expressed as μg β-carotene/g annatto seed using a previously prepared β-carotene standard curve (0.06–0.46 μg mL^−1^) with a determination coefficient of 0.994.

Norbixin content was estimated using the method described by [30]. NE and EE were diluted 10,000 times in 0.5% (*w*/*v*) potassium hydroxide solution. The absorbance was obtained using a UV-Vis spectrophotometer at 482 nm, and the norbixin content (%) was calculated with Equation (4).

(4) $$Norbixin\;content\;\left(\%\right)=\frac{Abs}{2870}\times \frac{100000}{sample\;weight\;\left(\mathrm{mg}\right)}\times 100$$

where A is the absorbance at 482 nm and 2870 is the norbixin absorptivity coefficient (g 100 g^−1^).

#### 2.3.5. Antioxidant Activity

The antioxidant activity of NE and EE was determined by the ABTS free radical scavenging (ABTS) method [31] and the Ferric reduction antioxidant power (FRAP) method [32,33]. The results were expressed as mg Trolox equivalent g^−1^ annatto seed extract using a previously prepared Trolox standard curve (0.02–0.38 mg mL^−1^) with a determination coefficient of 0.996. For the FRAP method, the results were expressed as mg Trolox equivalent g^−1^ annatto seed extract using a previously prepared Trolox standard curve (0.02–0.22 mg mL^−1^) with a determination coefficient of 0.991.

### 2.4. Production and Characterization of Gelatin- and Starch-Based Inks

#### 2.4.1. Production of Gelatin- and Starch-Based Inks

Gelatin-based hydrogels were used as ink for 3D food printing, according to Yap et al. [9], with modifications. Three treatments were studied: control gelatin-based hydrogel (Ge-C) with CC:LA (Ge-CC:LA) and NE (Ge-NE). First, the gelatin was hydrated in distilled water for 30 min (10 g gelatin/100 g suspension, dry basis) at room temperature, and then it was heated at 55 °C for 15 min using an ultra-thermostatic bath (MA-184, Marconi, Piracicaba, SP, Brazil) for solubilization. For Ge-CC:LA and Ge-NE, CC:LA or NE (25 g/100 g gelatin), respectively, was added under mechanical stirring at 350 rpm (mechanical stirrer RW20 digital, IKA^®^, Campinas, SP, Brazil) for 10 min at 40 °C.

Starch-based hydrogels were also used as ink for 3D food printing. Three treatments were studied: control starch-based hydrogel (St-C), with CC:LA (St-CC:LA) and NE (St-NE). Starch suspensions (10 g starch/100 g suspension, dry basis) were gelatinized using an ultra-thermostatic bath at 85 °C under mechanical stirring at 350 rpm for 30 min. For St-CC:LA and St-NE, CC:LA or NE (25 g/100 g starch), respectively, was added under mechanical stirring at 350 rpm for 10 min at 40 °C.

The gelatin-based hydrogel forming solutions and starch pastes were transferred to 10 mL syringes, cylindrical plastic molds, or cuboid molds. Syringes and molds were stored at 4 °C for 24 h at 100% relative humidity. Subsequently, the syringes were used for 3D printing and rheological behavior analysis, the cylindrical plastic molds for firmness and adhesiveness assay, and the cuboid molds for color measurements, texture profile analysis, and fork pressure test.

#### 2.4.2. Firmness and Adhesiveness

Ge- and St-based hydrogels were analyzed for firmness and adhesiveness after 24 h of storage in cylindrical plastic molds measuring 26.5 mm × 26.5 mm (diameter × height) at 4 °C with 100% relative humidity. Prior to analysis, the Ge-based hydrogels were kept at 25 °C for 1 h. The analysis was carried out with a puncture assay using a texturometer (TA.XTplusC, Stable Micro Systems Ltd., Godalming-Surrey, UK) with a load cell of 50 kgf (490 N), according to Maniglia et al. [14] and Sponchiado et al. [6].

The Ge- and St-based hydrogels were penetrated 10 mm in height with a 5 mm diameter cylindrical probe (P/0.5) at 1 mm s^−1^. The firmness of the hydrogels was obtained as the maximum energy required to penetrate the hydrogels (N), and the adhesiveness was calculated by the area under the curve of force versus penetration distance (mJ).

#### 2.4.3. Rheological Behavior

The rheological behavior of Ge- and St-based hydrogels was analyzed using a controlled stress rheometer with a Peltier system for temperature control and equipped with a cone-plate geometry (cone angle 2°, 40 mm diameter, and gap 55 μm) at a temperature of 25 °C for St-based hydrogels [6]. Specifically for Ge-based hydrogels, a temperature scanning test was previously carried out to determine the temperature of the rheological tests based on the sol–gel and gel–sol transition temperatures, as it is a thermoreversible physical hydrogel [34]. The temperature scanning test was performed by cooling the Ge-based hydrogels from 40 to 5 °C and immediately heating them from 5 to 40 °C, at a rate of 2 °C min^−1^, frequency of 1 Hz, and strain of 1%. The sol–gel and gel–sol transition temperatures were determined by the first derivative of the storage modulus (G′) versus temperature curve (Figure S1, Supplementary Materials). Based on these results, it was decided to carry out the rheological tests of Ge-based hydrogels at 20 °C (dynamic oscillatory measurements) and 40 °C (steady-state flow measurements).

Steady-state flow measurements were performed by a one-cycle shear after 40 s of soak time and 5 s of equilibration, with shear rates ranging from 1 to 1000 s^−1^. The results were obtained from curves of apparent viscosity versus shear rate. For Ge-based hydrogels, Newtonian behavior was observed. The power-law model (Equation (5)) was suitable for fitting the flow curve data (R^²^ > 0.99) of St-based hydrogels, where *η* is the apparent viscosity (Pa s), *K* is the consistency index (Pa s^n^), $\dot{\gamma }$ is the shear rate (s^−1^), and *n* is the flow behavior index (dimensionless).

(5) $$\eta =K\;{\dot{\gamma }}^{n-1}\;$$

For dynamic oscillatory measurements, the region of viscoelasticity of the Ge- and St-based hydrogels was determined by amplitude oscillatory strain test, with the oscillation strain varying from 0.100 to 1000% with a fixed frequency of 1 Hz. Then, the strain amplitude was fixed at 1% for both Ge- and St-based hydrogels (viscoelastic region), and the frequency sweep test was performed by varying the frequency from 0.01 to 16 Hz. The results were presented as G′ (storage modulus) and G″ (loss modulus) versus oscillation strain. The loss factor, expressed as tan δ = G″/G′, was calculated with data from the curve of G′ and G″ versus oscillation strain in the plateau region. The thixotropy was determined by a strain sweep test, alternating the shear rate between 0.1 and 100 s^−1^ every 10 s, totaling 5 flow peak holds. The percentage of shear recovery rate was calculated by the ratio between the viscosity of the fifth and first stages [8].

### 2.5. Production and Characterization of 3D Printed Gummy Based on Gelatin and Starch

#### 2.5.1. Production of 3D Printed Gummies

The Ge- and St-based formulations were 3D printed using a 3D bioprinter (bioV4-BioEdPrinter v4-4 Modular extrusion type of BioEdTech (São Paulo, SP, Brazil)). The cuboids (15 mm × 15 mm × 7.5 mm), unfilled stars (42 mm × 42 mm), and bear-shaped gummies (30 mm × 42 mm × 8 mm) were 3D printed at room temperature [6]. For Ge-based formulations, the following pre-optimized parameters were used: robotic arm speed of 150 mm/min, extrusion flow of 45 mm³/s, needle diameter of 0.41 mm, and filling percentage of 100%. For St-based formulations, these parameters were: robotic arm speed of 50 mm/min, extrusion flow of 60 mm³/s, needle diameter of 0.63 mm, and filling percentage of 100%. Bear-shaped gummies were used for visual analysis. Cuboid-shaped gummies were conditioned at 4 °C and 100% relative humidity for 24 h before color, printability, texture, and fork pressure tests. Unfilled stars were also used to determine the printability parameters. Molded gummies were also produced in the shape of cuboids and bears as controls.

#### 2.5.2. Printability and Reproducibility

Photographs of the surface cuboids and stars of all Ge- and St-based gummies were captured in triplicate using an iPhone 13 camera (Apple Inc., Cupertino, CA, USA), and the images were analyzed by ImageJ software (version 1.54 k, 25 February 2025). The area of the surface cuboids and the angle of the stars were calculated to assess the printability and shape reproducibility. The percentage of geometric fidelity rate was calculated by the ratio between the area of the 3D printed cuboid and the area of the model, and by the ratio between the angle of the 3D printed star and the angle of the star model [6].

#### 2.5.3. Color and Antioxidant Activity

The color parameters (L*, a*, and b*), chroma (C*), and hue angle (h*) of Ge- and St-based 3D printed gummies were obtained using a colorimeter, according to the method described in Section 2.3.2. using a measuring opening of 6 mm.

#### 2.5.4. Texture Profile Analysis

The texture profile analysis (TPA) of 3D printed and molded Ge- and St-based gummies was obtained using a texturometer with a load cell of 50 kgf (490 N), according to Renaldi et al. [10] with slight modifications. Test specimens were 3D printed or molded in the shape of cuboids measuring 15 mm × 15 mm × 7.5 mm (length × width × height). The TPA was conducted at room temperature, using a 40 mm diameter cylindrical probe (P/40), carrying out two cycles of compression and decompression. The TPA parameters were as follows: pre-test speed of 1 mm s^−1^, test speed of 5 mm s^−1^, post-test speed of 5 mm s^−1^, a strain of 75%, trigger force of 0.049 N, and delay between the two compressions of 5 s. The results of TPA were obtained from force (N) versus time (s) curves.

#### 2.5.5. Fork Pressure Test

The fork texture test was carried out to analyze whether gummies based on Ge- and St-based hydrogels meet the needs of people with swallowing and chewing difficulties (dysphagia), according to the standards established by the IDDSI—International Dysphagia Diet Standardization Initiative [35]. The fork pressure test was used to classify the gummies according to IDDSI consistency levels. The test consisted of pressing each formulation using the thumb on the base of a fork until the thumbnail turned white, since the force applied reflects the pressure of the tongue during swallowing. The test was carried out on 3D printed and molded cuboids measuring 15 mm × 15 mm × 7.5 mm (length × width × height), after 24 h of storage at 5 °C and 100% relative humidity by the same person (Sara Balabram).

### 2.6. Statistical Analysis

All characterizations were performed at least in triplicate and results were expressed as mean ± standard deviation. Data were analyzed using the TBICo Statistica^TM^ 13.0 (StatSoft GmbH, Hamburg, Germany) using one-way and two-way analysis of variance (ANOVA) and Tukey’s test at 5% significance (α = 0.05).

## 3. Results and Discussion

### 3.1. Characterization of NADES

The pH is an important parameter for the general application of NADESs, as it is a crucial factor for biochemical reactions and directly affects the yield of extractions of bioactive compounds [36]. The pH values of CC:LA and etOH-60% were acidic (<7.0), with the pH value of CC:LA being lower (*p* < 0.05) than that of etOH-60% (Table 1). The pH value of the NADES is mainly affected by the structure of the HBD [24], and lactic acid may have been mainly responsible for the low pH value of CC:LA. In general, pH values < 4.0 show better extraction efficiencies for bioactive compounds [37].

**Figure 1:**
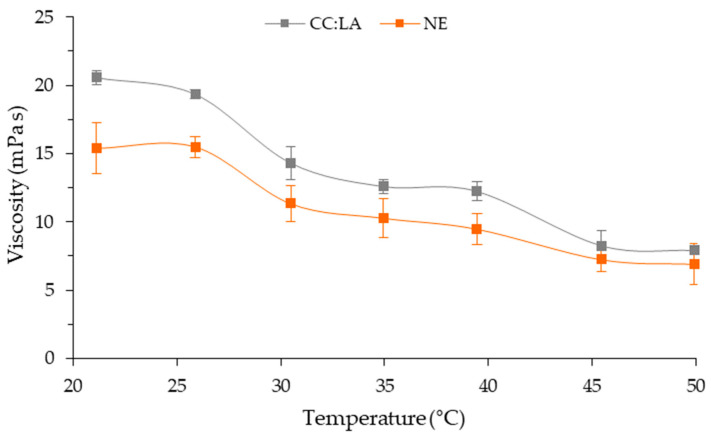
Curves of viscosity at 1s^−1^ versus temperature ranging from 20 to 50 °C of NADES based on choline chloride–lactic acid (CC:LA) and NADES–annatto seed extract (NE).

The viscosity of NADES can be considered one of the main physicochemical characteristics, as it can significantly affect the extraction yield of bioactive compounds [38], and is affected by the nature of the components and the amount of water. NADESs with high viscosity decrease the molecular movement and mass transfer, reducing solid–solvent interaction and, consequently, extraction yield [20,38]. NADESs based on choline chloride–lactic acid more efficiently extracted bioactive compounds from blueberry extract than those produced with choline chloride and citric acid, malonic acid, or tartaric acid [38]. The same behavior was observed for pomegranate peel extracts, with NADES based on choline chloride–lactic acid showing a higher extraction yield of bioactive compounds compared to conventional solvents [26]. In both cases, NADESs based on choline chloride–lactic acid presented the lowest viscosities than other formulations. The apparent viscosity of CC:LA decreased with increasing temperature (Figure 1), as observed by Alcade et al. [39] and Fanali et al. [36]. At 25 °C, the apparent viscosity of CC:LA was greater than that of water (≈0.89 mPa s) and ethanol (≈1.08 mPa s) [24] but similar to that of the NADES of the same formulation [26,36]. This higher viscosity of the NADES is caused by the strong intermolecular interactions between HBA and HBD (e.g., hydrogen bonds, van der Waals, and electrostatic interactions), resulting in lower molecular mobility, in addition to the “hole theory”, which are empty spaces formed between the constituents during the formation of NADESs [24].

Finally, another characteristic of NADESs that directly affects the extraction yield and the profile of bioactive compounds is polarity. Polarity can be estimated indirectly by the solvatochromic colorimetric method, which evaluates changes in the E_NR_ of the Nile Red dye diluted in the solvent [25]. The E_NR_ parameter obtained by Equation (1) is related to the polarity of the solvent, with more non-polar solvents having higher values of this parameter. CC:LA had a lower E_NR_ value (*p* < 0.05) than etOH-60%, indicating that it is more polar. Both the E_NR_ values of CC:LA and etOH-60% were similar to those reported in the literature [25,40], with the E_NR_ value of pure water being close to that of CC:LA (48.2 to 48.9 kcal mol^−1^) [40].

### 3.2. Characterization of NADES–Annatto Seed Extract

NE showed different properties compared to EE (Table 2), a control organic solvent for the extraction of annatto seed compounds. The pH value of NE was lower (*p* < 0.05) than that of EE, which was already expected, since CC:LA was more acidic than etOH-60% (Table 1). The NADES based on choline chloride–lactic acid had a pH value at 25 °C of 1.44, while the pH value of pomegranate peel extract produced with this NADES was 2.27 [26]. It indicates that the pH value depends mainly on the pH of the solvent used for extraction, the plant species, and the chemical composition of the extract.

As for CC:LA, the apparent viscosity of NE decreased with increasing temperature (Figure 1). Up to 40 °C, NE showed lower apparent viscosity than the CC:LA. This may occur due to the presence of bioactive compounds in the annatto seed, which may interfere with the intermolecular interactions between choline chloride and lactic acid, increasing the mobility of the NADES structure formed. Above 45 °C, no differences were observed between the apparent viscosities of CC:LA and NE.

**Figure 2:**
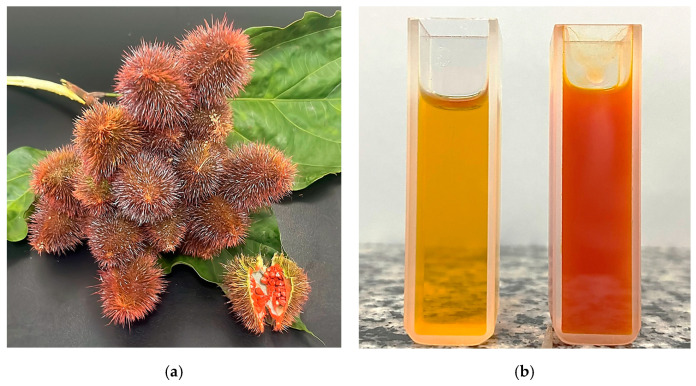
Photograph (**a**) of the fruits and seeds of the annatto tree (*Bixa orellana*) and (**b**) NADES–annatto seed extract (left) and ethanolic annatto seed extract (right).

The main bioactive compound in annatto seeds is bixin (*cis*-bixin), which corresponds to almost 80% of the carotenoids present, depending on the plant origin, maturation time, and post-harvest conditions [17]. Other carotenoids can be found in annatto seeds, mainly norbixin and isobixin, as well as β-carotene in smaller quantities. Geranylgeraniol, which is an active diterpenoid, can also be found in large quantities in annatto seeds [17]. But bixin and norbixin are the main pigments responsible for the intense reddish-orange color of annatto seeds (Figure 2a) [16].

The bixin content and total carotenoid content in NE were lower (*p* < 0.05) than those found in the EE (Table 2). In the case of EE, the bixin content was approximately 71% of the total carotenoids extracted. Bixin and carotenoids, in general, are fat-soluble pigments [20], which makes them more easily extracted by organic solvents than aqueous solvents. As etOH-60% showed a higher E_NR_ value than CC:LA, its extraction yield of bixin and other carotenoids from annatto seeds was more efficient. In the case of NE, bixin corresponded to 45% of the total carotenoids extracted, possibly because there was greater extraction of norbixin, which is a more water-soluble carotenoid than bixin [17]. The bixin content of EE was higher than the value of 260 μg bixin g^−1^ annato seed reported in the literature for extraction using ethanol–water 1:1 [41]. No research was found in the literature using NADES for the extraction of bixin and other carotenoids from annatto seeds, which represents the innovation of this work.

Although EE has higher bixin and total carotenoid contents, the antioxidant activity between both NE and EE, determined by the ABTS and FRAP methods, was similar (*p* > 0.05). Although carotenoids are the most abundant bioactive compounds in annatto seeds, other types of bioactive compounds can also be found. Some terpenoids, in addition to geranylgeraniol and amino acids, may be part of the annatto seed composition in different proportions, depending on the plant source [17]. Furthermore, phenolic compounds have already been identified in large quantities in annatto seed extracts [41] and annatto residues [21], with antioxidant activity values similar to NE. Certainly, by extracting other classes of more hydrophilic bioactive compounds, such as phenolic compounds, NE presented antioxidant activity similar to EE. Unfortunately, the Folin–Ciocalteu reagent reduction method, which estimates the content of phenolic compounds and other reducing agents, cannot be used for choline chloride-based NADESs, as precipitation of the Folin–Ciocalteu reagent occurs.

The color results were also consistent with bixin and total carotenoid content (Table 2). Visually, NE was orange and more translucent, while EE was redder and less translucent (Figure 2b). Physically, the measured color parameters were different between the two extracts, and visual differences could be measured. Both NE and EE presented the a* and b* parameters with positive values, indicating that they tended to be more yellow and red, respectively. The NE showed higher L* and b* values (*p* < 0.05), which means that it was lighter and more yellowish than the ethanolic annatto seed extract.

Furthermore, NE had a more saturated color (higher C* value) and an orange hue (indicated by a h* value of around 50°) than the EE (*p* < 0.05), which had a less vibrant color and a more reddish hue [23]. Similar values of h* of the EE were reported for annatto seed extracts produced with methanol, ethanol, and ethyl acetate (~26.5°) [22], while aqueous annatto seed extracts presented h* values like those of the NE (44.4°) [18]. This difference may be related to the difference in bixin content, which has an intense reddish-orange color [22]. A higher bixin content, as is the case in the EE (Table 2), tends to reduce the h* value, which can be confirmed by quantifying bixin by HPLC, where the EE presented a higher (*p* < 0.05) concentration of bixin content.

### 3.3. Characterization of Gelatin- and Starch-Based Inks

#### 3.3.1. Firmness and Adhesiveness

**Figure 3:**
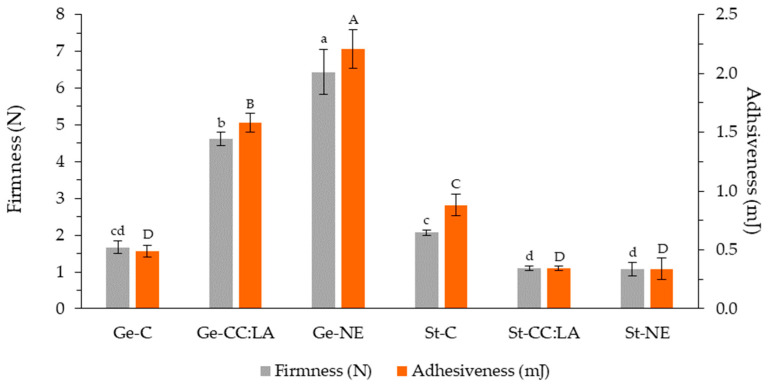
Maximum peak force (expressed as firmness) and adhesiveness of gelatin (Ge)- and starch (St)-based hydrogels without (C) and with NADES based on choline chloride–lactic acid (CC:LA) or NADES–annatto seed extract (NE). Different ^a–d^ lowercase and ^A–D^ uppercase letters indicate significant differences in firmness and adhesiveness, respectively, according to Tukey’s test (*p* < 0.05).

For the firmness and adhesiveness results, the addition of CC:LA and NE impacted the mechanical and rheological properties of the Ge- and St-based inks in distinct ways (Figure 3). For Ge-based inks, firmness and adhesiveness were increased in the presence of CC:LA and NE, showing values higher (*p* < 0.05) than those observed for Ge-C. Furthermore, Ge-NE presented the highest values for both parameters (*p* < 0.05). This suggests that the bioactive compounds in the NE may have a more pronounced structuring effect than the CC:LA, possibly due to additional interactions with the gelatin network [8]. Superior values of firmness have been reported for gelatin-based hydrogels with concentrations of 5% and 10.7% (4 N and 14 N, respectively) [15].

On the other hand, St-based inks exhibited the opposite behavior. The St-C showed higher firmness and adhesiveness compared to the inks containing CC:LA and NE (*p* < 0.05), suggesting that the addition of these components weakened the starch hydrogel network and made it more flexible [42]. Furthermore, no differences were observed between the firmness and adhesiveness values of St-CC:LA and St-NE (*p* > 0.05), indicating that both systems interfere with the hydrogel structure in a similar way. Overall, Ge-C, St-CC:LA, and St-NE presented the lowest firmness and adhesiveness values (*p* < 0.05). The firmness of hydrogels with 5 and 10.7% unmodified and thermally modified cassava starch, without or with the addition of 20% gelatin, varied between 0.5 N and 2 N, values close to those reported for St-based inks [15].

These results demonstrate that the influence of CC:LA and NE on the mechanical properties of the inks strongly depends on the matrix used. While the CC:LA reinforced the gelatin network, promoting an increase in firmness and adhesiveness, in the starch matrix, both CC:LA and NE contributed to the reduction in these properties. This behavior can be explained by the difference in the chemical and structural nature of the macromolecules involved: gelatin, a protein, is more susceptible to forming structured networks through interactions with CC:LA and bioactive compounds, whereas starch, a polysaccharide, may undergo a plasticizing effect due to the presence of compounds that interfere with its intermolecular bonds [43].

From the perspective of applying these hydrogel-based inks in food formulations for people with dysphagia, reducing firmness and increasing flexibility are desirable as they make food easier to chew and swallow [44]. Studies indicate that the texture of food for individuals with dysphagia should be adjusted to ensure safe swallowing, with hydrogels recommended to provide adequate viscosity without compromising nutritional composition [2,5,15]. Formulations that reduce the rigidity of hydrogel-based inks and provide flexibility may be advantageous in the development of products for this population. On the other hand, excessive reduction in firmness can compromise food handling and palatability, impacting consumer acceptance [5]. Therefore, optimizing the formulation of these hydrogel-based inks should consider a balance between softness and viscosity to ensure both safety and acceptability of the products. In this case, the St-CC:LA and St-NE inks presented ideal firmness for the development of foods for dysphagic patients, which must have a maximum value of 1.5 N [15].

#### 3.3.2. Rheological Behavior

**Figure 4:**
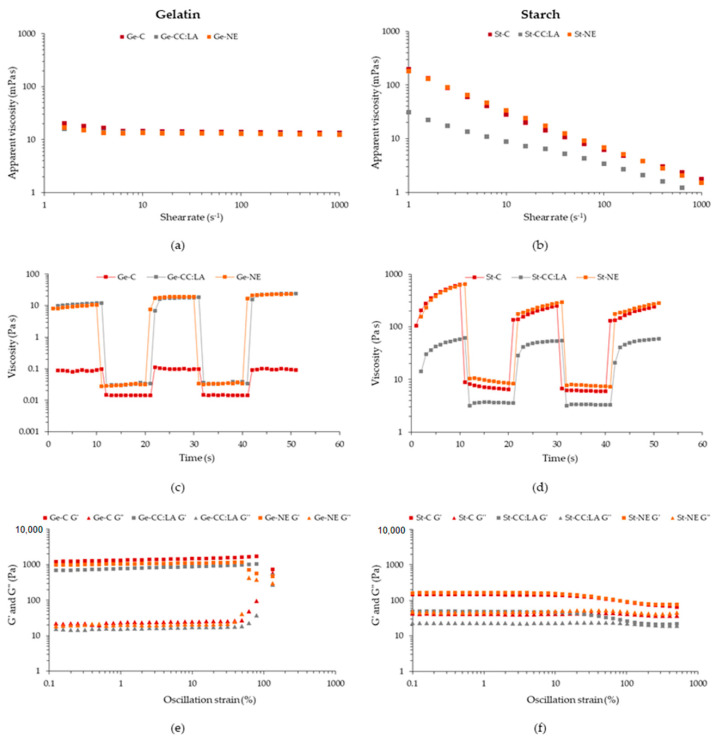
Flow curves (**a**,**b**), structure recovery profiles (**c**,**d**), and storage (G′) and loss (G″) moduli versus oscillation strain (**e**,**f**) of gelatin (Ge)- and starch (St)-based hydrogels without (C) and with NADES based on choline chloride–lactic acid (CC:LA) or NADES–annatto seed extract (NE).

In the extrusion 3D printing process, when the material is forced through a very fine nozzle, it is subjected to a high level of shear [13]. Therefore, it is essential to know the behavior of the material during shear. Viscosity is a crucial rheological parameter that ensures continuous flow during 3D printing [11]. The apparent viscosity as a function of shear rate of Ge- and St-based inks was affected by the type of biopolymer and the incorporation of CC:LA and NE (Figure 4a,b).

All Ge-based inks, at low shear rate, showed slight variability in apparent viscosity, probably related to the sensitivity of the equipment with low viscosity materials. From a shear rate of 6.3 s^−1^, the Ge-based inks, without or with CC:LA or NE, showed a Newtonian fluid behavior, with the apparent viscosity remaining practically constant even with an increase in the shear rate (Figure 4a). The addition of CC:LA or NE did not modify the flow behavior of Ge-based inks, and no differences (*p* > 0.05) were observed between the apparent viscosities at 100 s^−1^ of Ge-C, Ge-CC:LA, and G-NE, which remain at around 13.2 mPa s (Table 3). This behavior of Ge-based inks can be influenced by different factors, such as gelatin concentration, temperature (since it is a thermoreversible physical hydrogel), and the gelatin type and source. At 40 °C, gelatin is in its sol domain, behaving as a low viscosity solution and with the effect of water–macromolecule interactions more pronounced than macromolecule–macromolecule or macromolecule–other components interactions [34]. In another study, for 5% type B bovine gelatin-based inks at 25 °C, the flow behavior was similar [12]. At low shear rates, the apparent viscosity decreased, and from 40 s^−1^ onwards, the behavior observed was that of a Newtonian fluid. This can be explained by the orientation and line-up of the gelatin chains in the direction of flow [12]. The temperature of 40 °C was chosen for the flow curves because, at the printing temperature (25 °C), the gelatin is in the region where it is in the transition between sol and gel and vice versa (Figure S1, Supplementary Materials), and the tests end up not being reproducible. Therefore, only the effects of CC:LA and NE in Ge-based inks were evaluated, because despite the low viscosity at 40 °C, at 25 °C, the 3D printing of food was successful, as will be observed in the next section.

On the other hand, St-C, St-CC:LA, and St-NE exhibit pseudoplastic-type non-Newtonian fluid behavior (Figure 4b). This means that the apparent viscosity decreased with the increase in the shear rate, showing a shear-thinning behavior, which can be confirmed by the power-law model adjustment parameters (R^²^ > 0.99), where *n* < 1 indicates a pseudoplastic fluid [11]. Shear thinning behavior is typical of some macromolecular fluids, such as starch, as it occurs because of the alignment and orientation of polymer chains under shear force [13]. This behavior is typical for starch-based inks [6,11,45] and can be desirable for 3D printing by extrusion as it facilitates control of layer deposition and increases precision, while also improving the maintenance of the structure of the deposited material [6]. As an effect of the addition of CC:LA or NE, CC:LA reduced (*p* < 0.05) the value of *K*, *n* and the apparent viscosity at 100 s^−1^ in relation to St-C and St-NE, while NE increased (*p* < 0.05) these parameters in relation to St-C and St-CC:LA. NADESs, such as CC:LA, can have a plasticizing effect on starch-based hydrogels [43], reducing intermolecular interactions between starch chains and, consequently, increasing fluidity and flexibility. NE, on the other hand, can interact with starch chains due to the presence of bioactive compounds (e.g., carotenoids, terpenoids, and phenolic compounds), increasing the apparent viscosity and flow resistance of the inks. The same trend was observed for hydrogels based on corn starch and/or chitosan incorporated with murta leaf extract [45]. *K* values below 0.7 have been reported as positive for 3D printing, as higher values can cause discontinuity in the ink lines and difficult printing [4]. All St-based inks presented desirable *K* values for good 3D printing.

In addition to steady-state flow measurements, the thixotropy test provides important information about the behavior of the ink during 3D printing. In extrusion 3D printing, the material is subjected to different levels of shear: in the storage syringe, there is low shear, while in the nozzle, the shear is high [4]. For good extrusion, the material must be easily extruded and maintain its mechanical integrity, requiring rapid structural recovery after extrusion [11]. Studying the viscosity of biopolymer-based inks under different shear rates is essential to understand their thixotropic properties and evaluate the loss and recovery of the biopolymer structure [13].

Both Ge- and St-based inks showed a high decrease in viscosity under high shear, with viscosity recovery under low shear (Figure 4c,d). Although the Ge-based inks exhibited Newtonian fluid behavior, a typical thixotropic behavior was observed, possibly because the low shear rate was performed at 0.1 s^−1^, a region in which the viscosity of the Ge-based inks varied (<6.3 s^−1^). The viscosity of Ge-based inks at low and high shear remained stable and recovered rapidly at low shear, indicating an instant recovery of the structure, which is desirable for 3D printing to improve the geometric accuracy and self-support capacity of the printed food [7]. St-based inks, on the other hand, showed more pronounced thixotropic behavior, requiring more time to recover viscosity at low shear, which can reduce the accuracy of 3D printing. The addition of CC:LA decreased the viscosities at low and high shear for St-CC:LA, while the addition of NE recovered the viscosity of the St-NE. For Ge-based inks, the incorporation of both CC:LA and NE increased the viscosities at low and high shear for Ge-CC:LA and Ge-NE.

Thixotropic behavior can be explained by the theory of polymer conformation, with the ability of the chains to reorganize due to the self-healing property of the physical cross-links in the matrix structure [13]. Viscosity decreases under high shear due to the orientation of the biopolymer chains in the direction of flow, reducing the conformational entropy of the system [11]. The conformational entropy is fully or partially recovered when shear decreases, and biopolymer chains recover their interconnected structure. This same behavior was also observed for inks based on cassava, potato, and rice starch [11] and inks based on gelatin with or without phycocyanin [8]. Based on the shear recovery rate (Table 3), it was observed that the Ge-based inks presented values above 100%, which suggests that these inks have a high and rapid capacity for structural recovery, with a more structured reorganization after the application of shear. The incorporation of CC:LA and NE intensified this structuring, especially NE (*p* < 0.05), possibly due to the interactions that bioactive compounds can form in the biopolymeric network [8]. For St-based inks, the addition of CC:LA increased (*p* < 0.05) the shear recovery rate to above 90% compared to St-C and St-NE (≈40%). St-based inks showed a lower recovery capacity than Ge-based inks [7], which was expected, since gelatin forms a thermoreversible physical hydrogel, with a high capacity for structural reorganization. Although low shear recovery rates may indicate lower geometric accuracy and self-support, this could be an interesting property to form 3D printed foods based on weaker hydrogels and suitable for people with dysphagia.

Food inks for 3D printing must not only have adequate viscosity and thixotropy for efficient extrusion through the nozzle but also have G′ values (storage modulus) high enough to guarantee the structural strength of the 3D printed food [4]. In oscillatory rheology, samples are subjected to increasing oscillating deformations (strain sweep) at a constant frequency, or at a decreasing frequency with constant strain (frequency sweep), within the linear viscoelastic range [13]. The storage modulus (G′) measures the energy stored and recovered by the material per cycle, reflecting its solid or elastic behavior, and the loss modulus (G”) indicates the energy dissipated, characterizing the liquid or viscous behavior [6,13]. The combination of these two parameters provides crucial information about the viscoelasticity of polymeric solutions and the post-deposition quality of the 3D printed food [11].

For all Ge- and St-based inks, G′ was greater than G″ throughout the oscillation strain (Figure 4e,f), indicating an elastic behavior [4]. This means that these hydrogel-based inks are more resistant to deformation and maintain their shape, ensuring the consistency and stability of the 3D printed food. All Ge- and St-based inks also showed tan δ value < 1 in the plateau region, indicating that they can respond to elastic deformation and support the 3D printing layers, with a more resistant structure [11]. However, the G′ values of Ge-based inks were much higher than those of St-based inks (Figure 4e,f), and, consequently, the tan δ values were lower, indicating a more solid-like structure [46]. Tan δ values between 0.1 and 0.3 were reported for weak hydrogels with ideal rheological characteristics for good 3D printing and for printed food for people with dysphagia [6,46]. In this sense, St-based inks presented more suitable rheological parameters for this purpose.

### 3.4. Characterization of 3D Printed Gummies Based on Gelatin and Starch

#### 3.4.1. Printability and Reproducibility

**Figure 5:**
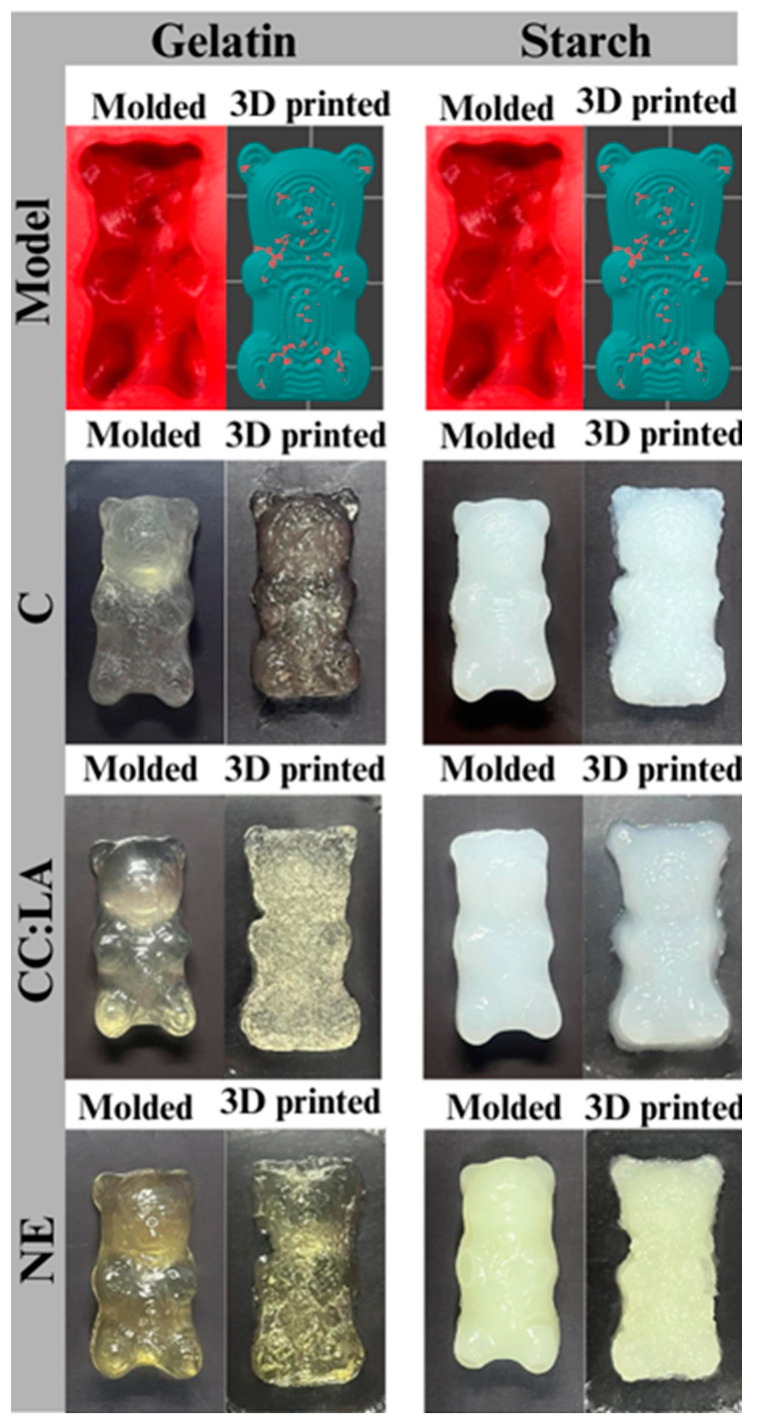
Photographs of 3D printed and molded bear-shaped gummies based on gelatin (Ge)- and starch (St)-based hydrogels without (C) and with NADES based on choline chloride–lactic acid (CC:LA) or NADES–annatto seed extract (NE).

One of the benefits that 3D food printing provides is the possibility of producing foods with different shapes and textures [4]. For people with dysphagia, who require food with a personalized texture, the visual appearance of printed food can be a positive factor [3]. Bear-shaped gummies based on Ge- and St-hydrogels, without or with CC:LA and NE, were 3D printed to show the possibility of producing printed foods with different shapes and good quality compared to the same molded formulations (Figure 5). Although the bear-gummies have the same formulation, the different production method can result in an alteration in the shape of the product obtained. For people with dysphagia, bear-shaped gummies are interesting because they are visually attractive and provide food with different shades of color and texture, which expands the possibilities for producing personalized foods.

For physical analysis, all formulations were 3D printed into cuboid and star shapes, and their pictures were used to evaluate printability (Table 4). Ge-based 3D printed cuboids showed greater geometric fidelity (*p* < 0.05) than St-based formulations, when compared to a geometric model. The addition of NE improved the geometric fidelity of St-NE to a value similar (*p* > 0.05) to Ge-based formulations. These results agree with the rheological properties, where it was observed that the St-based formulations, which presented a lower shear recovery rate and lower G′ values than the Ge-based inks, presented lower self-support after 3D printing, increasing the cuboid area in relation to the model.

Furthermore, the St-NE ink showed higher *n* and *K* values and better geometric fidelity. Regarding the other parameters studied, it is observed that the St-NE was the one that stood out the most among all the gummies, presenting positive characteristics for the 3D printing process. Although the Ge-based gummies presented greater geometric difficulties, their printed lines were not smooth and continuous, possibly due to the very solid-like behavior (low tan δ value) [46].

Observations like the results of cuboids were obtained for star-shaped 3D printing, which, for the St-NE gummy, presented the best geometric fidelity (*p* < 0.05) in relation to the star angle when compared to the 3D model. All the parameters evaluated were also positive for St-NE gummy. It can be said that the addition of NE did not favor the Ge-NE but was very positive in improving the printability of the St-NE gummy. This behavior can be explained through the rheological properties and mainly by the value of the tan δ parameter, which was higher for the Ge-based gummies in relation to the St-based gummies. Overall, St-NE presented rheological properties more suitable for 3D printing by extrusion and, consequently, better printability of different geometric shapes. In another study, hydrogels based on 5 and 10.7% cassava starch, unmodified or thermally modified, showed better printability parameters than hydrogels based on 5% gelatin [15]. Hydrogels based on 10% gelatin were not even suitable for 3D printing. Furthermore, a blend of 5% and 10.7% of gelatin and modified cassava starch improved printability parameters [15].

#### 3.4.2. Color

The color parameters were obtained for Ge- and St-based gummies, 3D printed and molded (Table 5). In relation to the different methods (3D printing and molding), corroborating what was shown in Figure 5, even in the case of the same formulation, the change in the production process caused changes in the color parameters, which can be observed in the visual aspect of the bear-shaped gummies.

The L*, a*, b*, C*, and h* parameters varied in different ways between the same formulation, comparing 3D printed and molded gummies, and between different 3D printed formulations. But, in general, the values of L*, a*, and b* were low (closer to zero), indicating that all samples were clear and in low light. The Ge-NE and St-NE gummies presented the more negative values of a*, which indicates a color tendency more towards green, and more positive values of b*, which indicates a more yellowish color tendency. Ge-C and Ge-CC:LA gummies also presented positive values of b*, which can be explained by the naturally yellowish color of the gelatin used. For Ge-NE and St-NE gummies, the yellowish and slightly greenish color is due to the addition of NE, which presented an orange color (Table 2).

All gummies also had a low C* value, which indicates that they were less saturated and vibrant, with softer and more neutral colors. For G-NE and St-NE, 3D printing increased (*p* < 0.05) the C* value in relation to molded gummies, while the addition of CC:LA did not affect (*p* > 0.05) this parameter between molded and 3D printed gummies. Furthermore, St-C gummy, followed by Ge-NE and St-NE gummies, presented higher (*p* < 0.05) C* values compared to the other 3D printed gummies. The increase in C* may be interesting for foods for people with dysphagia, as colorful foods tend to be more attractive [2]. The h* values, which indicate the hue, varied between yellow (around 90°) and greenish yellow (above 110°). Although St-CC:LA had a green h* value, visually they were white, making it difficult to determine this color from the h* value.

#### 3.4.3. Texture Profile Analysis

The TPA results (Table 6) showed that for the hardness parameter (4.17–77.61 N), there was a significant difference between all formulations when comparing the 3D printing method and the molded method; for 3D printing, all St-based gummies did not show differences between each other (*p* > 0.05) but were lower (*p* < 0.05) than Ge-C and Ge-CC:LA and Ge-NE, similar to each other (*p* > 0.05). Since the maximum force required to distort the gels on the first bite is directly related to hardness, the samples that present lower hardness values are favorable for use in foods for people with dysphagia, highlighting the 3D printing method applied to all tested St-based formulations and Ge-C gummy [10].

Regarding the adhesiveness parameter (1.94–50.41), no values were obtained for Ge-based gummies using the 3D printing method, only with the molded method, presenting an average value of 20.09. For St-based gummies, no difference in adhesiveness was observed between the same formulation using molded and 3D printing methods (*p* > 0.05), except for St-NE, which was the only gummy showing lower adhesiveness (*p* < 0.05) with the molded method compared to the 3D printing method. Additionally, all St-based gummies differed (*p* < 0.05) from each other for the adhesiveness parameter using the 3D printing method. Since adhesiveness is related to the lingual efforts made to propel the food bolus through the throat and the risk of choking, lower values are preferable for use in foods for people with dysphagia, with the 3D printing method standing out for most formulations [3].

Regarding elasticity (0.51–1.09 mm), almost all Ge- and St-based gummies showed differences between 3D printing and molded methods (*p* < 0.05), except for the St-C formulation. Additionally, when comparing different formulations using the 3D printing method, it is observed that all Ge- and St-based gummies do not differ from each other (*p* < 0.05), with the lowest values being obtained from St-based gummies using the 3D printing method. The cohesiveness was similar when comparing the various formulations using the two methods (*p* > 0.05), except for St-C gummy. However, when comparing the 3D printing method for the different formulations tested, no difference was recorded between the Ge-based gummy, whose mean cohesiveness value was 0.89 mJ. However, they differed from St-based formulations (*p* < 0.05), which also differed from each other. St-NE gummy using the 3D printing method presented the lowest cohesiveness value among the samples analyzed (*p* < 0.05), highlighting the positive influence of adding NE to the St-based gummy for this parameter, as it relates to the food’s ability to retain shape between the first and second compressions. The lower the cohesiveness, the more suitable the food is for use in diets for people with chewing difficulties [5].

For the gumminess parameter, all gummies showed a significant difference between 3D printing and molded methods, with the 3D printing method presenting lower gumminess (*p* < 0.05) for all gummies compared to the molded method. Three-dimensional printed Ge-based gummies did not differ from each other (*p* > 0.05), with an average gumminess value of 17 N, which was higher (*p* < 0.05) than the values observed for 3D printed St-based formulations (average value of 1.9 N). Since the gumminess measure describes the energy required to transform a semisolid food into a bolus capable of being swallowed, the lower the energy needed for this, the more favorable the food is for dysphagia people, thus highlighting the contribution of 3D printing in all tested gummies, with the best result obtained by St-NE (1.29 N) [2].

Therefore, through the joint analysis of the results obtained for hardness, adhesiveness, elasticity, cohesiveness, and gumminess parameters, it is possible to confirm that the 3D printing method is preferable for obtaining foods for dysphagia diets due to value reductions in most of the parameters when compared to the molded method. Additionally, St-based gummies showed better results compared to Ge-based gummies, and the addition of NE proved favorable for most of the considered parameters, with the St-NE gummy showing the best results for the intended purposes.

#### 3.4.4. Fork Pressure Test

**Figure 6:**
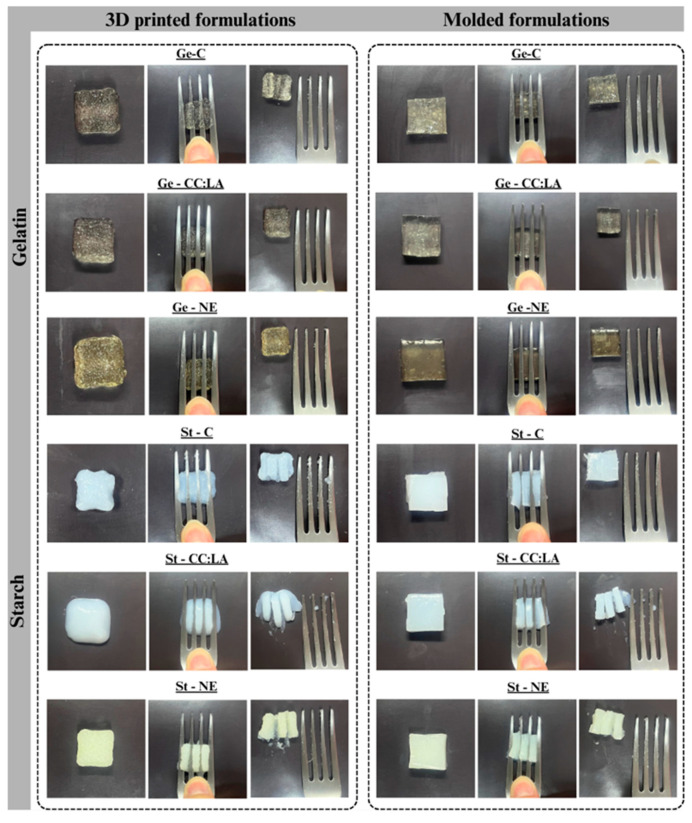
Photographs of IDDSI fork test on 3D printed and molded gummies formulations in cuboid shape based on gelatin (Ge)- and starch (St)-based hydrogels without (C) and with NADES based on choline chloride–lactic acid (CC:LA) or NADES–annatto seed extract (NE).

The fork pressure test by the IDDSI framework offers a comprehensive and practical assessment of modified food textures for patients with dysphagia [35,46]. The fork pressure test was performed using 3D printed gummies in cuboid shape with a size of 15 mm × 15 mm × 7.5 mm (length × width × height) and molded cuboids with the same dimensions (Figure 6), as these measurements are considered ideal to avoid choking risks in dysphagic patients [3]. The maximum pressure applied by the fork was controlled by the appearance of a clear tip on the thumbnail, which simulates the pressure exerted by the tongue (~17 kPa) during swallowing [46].

The results indicated that none of the gummies obtained, whether by the molded method or 3D printing, were able to meet all the parameters corresponding to level 5 established by the IDDSI (Table 7). However, a greater range of parameters was observed for the same 3D printed gummies compared to molded ones. By changing from the molded method to 3D printing, the Ge-C gummy went from meeting one parameter to four, while St-C, St-CC:LA, and St-NE gummies met two additional parameters with the 3D printing method. Ge-CC:LA and Ge-NE gummies showed no changes in parameters when comparing the molded method with 3D printing. Three-dimensional printing proves to be efficient in reducing firmness of St-based gummies compared to molded gummies [1]. Therefore, the gummies that met more parameters (4/5) of level 5, minced and moist, of the IDDSI framework and have the potential to be included in the diets of people with dysphagia are the Ge-C, St-CC:LA, and St-NE gummies obtained by the 3D printing method. This confirms the benefits of adding CC:LA and NE to St-based gummies in increasing the number of parameters needed for developing foods that can be included in diets of people with chewing and swallowing difficulties.

## 4. Conclusions

In conclusion, this study demonstrates the potential of using NADES-extracted annatto seed bioactive compounds in hydrogel formulations for 3D printed foods tailored for dysphagia patients. The integration of gelatin and starch as key structural components played a crucial role in achieving the desired textural properties, ensuring that the printed foods maintained both mechanical integrity and appropriate softness for safe consumption. Gelatin, as a biopolymer with exceptional gel-forming abilities, contributed to the viscoelastic behavior necessary for extrusion-based 3D printing, allowing for enhanced structural fidelity and printability. The inclusion of NADES extracts not only improved the nutritional profile by incorporating valuable bioactive compounds but also influenced the mechanical properties of the gels, making them more suitable for dysphagic diets. Additionally, the formulation adjustments impacted the adhesiveness and hardness of the final products, crucial factors for ensuring ease of swallowing. The findings highlight the promising synergy between green extraction techniques and advanced food processing, paving the way for innovative, functional foods with enhanced textural, nutritional, and sensory attributes. Future studies should explore the long-term stability and sensory acceptability of these formulations, further refining the potential applications of gelatin-based 3D printed foods for specialized dietary needs.

## Figures and Tables

**Table 1:** Physicochemical properties of NADES based on choline chloride–lactic acid (CC:LA) and 60% (*v*/*v*) ethanolic solution (etOH-60%).

Properties	CC:LA	etOH-60%
pH at 25 °C	1.28 ± 0.02 ^b^	6.27 ± 0.02 ^a^
Density at 25 °C (g cm^−3^)	1.145 ± 0.007 ^a^	0.873 ± 0.006 ^b^
Viscosity at 25 °C (mPa s)	19.4 ± 0.3	-
E_NR_ (kcal mol^−1^)	48.1 ± 0.1 ^b^	50.3 ± 0.1 ^a^

E_NR_: transition energy; λ_max_: maximum absorption wavelength. Means ± standard deviation (n = 3). ^a,b^ Different letters on the same line indicate significant differences between CC-LA and etOH-60% according to Tukey’s test (*p* < 0.05).

**Table 2:** pH, color parameters, total carotenoids, norbixin contents, and antioxidant activity of NADES (NE) or ethanolic (EE) annatto seed extracts.

Properties	NE	EE
pH at 25 °C	1.40 ± 0.03 ^b^	5.34 ± 0.10 ^a^
Viscosity at 25 °C (mPa s)	15.5 ± 0.8	-
L*	48.9 ± 0.8 ^a^	42.5 ± 0.8 ^b^
a*	15.3 ± 0.1 ^a^	15.6 ± 0.8 ^a^
b*	18.8 ± 2.3 ^a^	7.5 ± 1.1 ^b^
C*	24.3 ± 1.7 ^a^	17.4 ± 0.9 ^b^
h* (°)	50.7 ± 3.7 ^a^	25.6 ± 3.4 ^b^
Bixin content by HPLC (μg g^−1^ annatto seed)	20.56 ± 1.04 ^b^	448 ± 59 ^a^
Total carotenoid content (μg β-carotene g^−1^ annatto seed)	45.66 ± 2.34 ^b^	632 ± 38 ^a^
Norbixin (%)	1.08 ± 0.02 ^b^	3.14 ± 0.26 ^a^
ABTS method (mg Trolox equivalent g^−1^ annatto seed)	4.99 ± 0.62 ^a^	5.76 ± 0.79 ^a^
FRAP method (mg Trolox equivalent g^−1^ annatto seed)	53.28 ± 4.67 ^a^	67.50 ± 10.83 ^a^

Means ± standard deviation (*n* = 3). ^a,b^ Different letters on the same line indicate significant differences between NE and EE according to Tukey’s test (*p* < 0.05).

**Table 3:** Power-law model parameters, apparent viscosity, tan δ in the plateau, and shear recovery rate of gelatin (Ge)- and starch (St)-based hydrogels without (C) and with NADES based on choline chloride–lactic acid (CC:LA) or NADES–annatto seed extract (NE).

Hydrogel-Based Ink	Power-Law Model			Apparent Viscosity at 100 s^−1^ (Pa s)	Shear Recovery Rate (%)	tan δ (G″/G′)
	*K*	*N*	*R* ^²^			
Ge-C	-	-	-	(13.36 ± 0.4) ^d^ × 10^−3^	103.5 ± 1.0 ^c^	0.0170 ± 0.0004 ^b^
Ge-CC:LA	-	-	-	(13.24 ± 0.1) ^d^ × 10^−3^	208.3 ± 15.3 ^b^	0.0197 ± 0.0006 ^a^
Ge-NE	-	-	-	(12.95 ± 0.3) ^d^ × 10^−3^	242.4 ± 9.7 ^a^	0.0192 ± 0.0007 ^a^
St-C	151.8 ± 4.1 ^b^	0.672 ± 0.004 ^b^	0.991	6.29 ± 0.08 ^b^	37.7 ± 1.2 ^d^	0.2828 ± 0.0035 ^b^
St-CC:LA	28.1 ± 0.7 ^c^	0.481 ± 0.006 ^c^	0.997	3.32 ± 0.08 ^c^	93.5 ± 5.8 ^c^	0.4325± 0.0197 ^a^
St-NE	169.9 ± 1.2 ^a^	0.691 ± 0.002 ^a^	0.999	6.83 ± 0.04 ^a^	43.5 ± 0.4 ^d^	0.2805 ± 0.0069 ^b^

Means ± standard deviation (n = 3). ^a–d^ Different letters in the same column indicate significant differences between Ge- and St-based inks according to Tukey’s test (*p* < 0.05). *K*: consistency index (Pa s^n^), *n*: flow behavior index (dimensionless), *G*′: storage modulus, *G*″: loss modulus.

**Table 4:** Photographs of 3D printed gummies formulations in cuboid and star shapes, and printability parameters of gelatin (Ge)- and starch (St)-based hydrogels without (C) and with NADES based on choline chloride–lactic acid (CC:LA) or NADES–annatto seed extract (NE).

**3D Printed Cuboids**	** 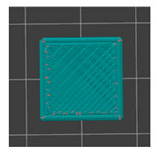 **	** 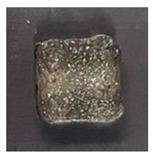 **	** 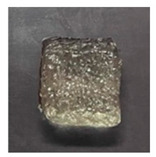 **	** 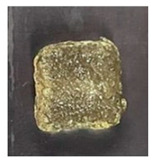 **	** 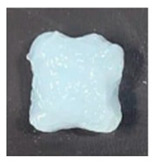 **	** 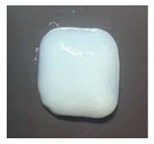 **	** 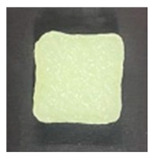 **
Formulation	Model	Ge-C	Ge-CC:LA	Ge-NE	St-C	St-CC:LA	St-NE
Area (cm^2^)	2.25	2.26 ± 0.02 ^b^	2.34 ± 0.18 ^b^	2.22 ± 0.01 ^b^	3.00 ± 0.08 ^a^	3.26 ± 0.37 ^a^	2.50 ± 0.12 ^b^
Geometric fidelity (%)	-	100 ± 1 ^b^	104 ± 8 ^b^	98 ± 1 ^b^	133 ± 4 ^a^	144 ± 16 ^a^	111 ± 5 ^b^
Smoothly and continuously extruded	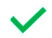	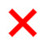	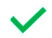	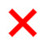	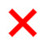	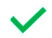	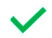
Keeps the structure after printing	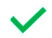	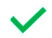	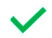	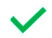	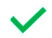	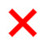	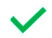
Smooth and continuous lines	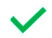	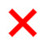	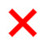	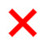	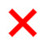	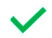	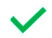
Without separation of lines	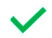	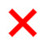	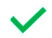	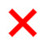	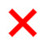	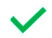	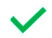
Higher resolution (well-defined shape)	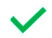	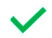	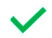	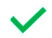	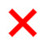	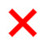	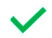
Broken deposited lines	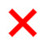	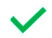	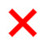	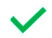	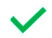	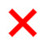	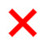
**3D printed stars**	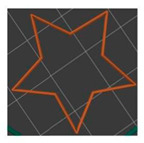	** 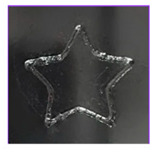 **	** 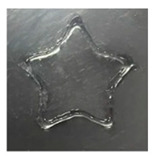 **	** 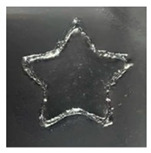 **	** 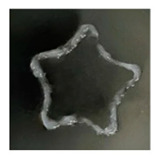 **	** 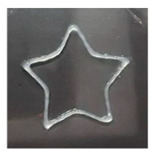 **	** 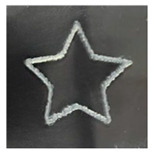 **
Formulation	Model	Ge-C	Ge-CC:LA	Ge-NE	St-C	St-CC:LA	St-NE
Star angles (°)	52	62 ± 3 ^cd^	65 ± 3 ^bc^	69 ± 1 ^b^	77 ± 0.8 ^a^	64 ± 2 ^bc^	56 ± 3 ^d^
Geometric fidelity (%)	-	119 ± 6 ^c^	125 ± 6 ^bc^	132 ± 2 ^b^	148 ± 1 ^a^	123 ± 4 ^bc^	108 ± 6 ^d^
Similar shape to the predesigned star geometry with well-defined angles	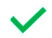	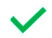	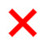	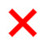	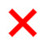	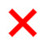	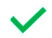
Continuous lines	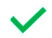	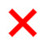	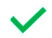	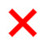	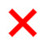	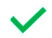	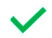
Retains its shape after printing	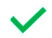	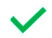	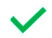	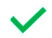	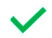	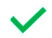	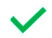
Smoothly extruded	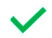	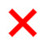	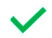	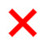	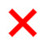	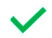	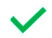

Mean ± standard deviation (*n* = 3). ^a–d^ Different letters on the same line indicate significant differences in printability parameters between 3D printed formulations, according to Tukey’s test (*p* < 0.05). Red cross: aspect not shown; green check: aspect shown.

**Table 5:** Color parameters of 3D printed and molded gummies formulations of gelatin (Ge)- and starch (St)-based hydrogels without (C) and with NADES based on choline chloride–lactic acid (CC:LA) or NADES–annatto seed extract (NE).

Formulation	Method	L*	a*	b*	C*	h*
Ge-C	3D printed	6.4 ± 0.3 ^cA^	−1.0 ± 0.1 ^cB^	3.4 ± 0.8 ^cA^	2.0 ± 0.8 ^dB^	104.6 ± 4.4 ^cA^
	Molded	7.9 ± 1.4 ^A^	2.4 ± 0.6 ^A^	4.4 ± 0.9 ^A^	5.4 ± 1.0 ^A^	63.1 ± 5.0 ^B^
Ge-CC:LA	3D printed	11.0 ± 0.1 ^aB^	1.0 ± 0.2 ^aA^	4.0 ± 0.1 ^cA^	4.1 ± 0.2 ^cA^	103.3 ± 3.7 ^cA^
	Molded	12.8 ± 1.1 ^A^	−1.1 ± 0.4 ^B^	4.0 ± 0.5 ^A^	4.2 ± 0.6 ^A^	104.9 ± 4.2 ^A^
Ge-NE	3D printed	10.0 ± 0.6 ^bA^	−0.7 ± 0.1 ^bcB^	7.8 ± 0.5 ^cA^	8.0 ± 0.8 ^bA^	94.7 ± 1.6 ^dB^
	Molded	11.4 ± 1.2 ^A^	−2.0 ± 0.3 ^A^	5.2 ± 0.6 ^B^	5.4 ± 0.8 ^B^	111.1 ± 5.4 ^A^
St-C	3D printed	3.1 ± 0.3 ^dA^	−0.4 ± 0.0 ^bB^	−10.3 ± 0.5 ^eB^	10.4 ± 0.5 ^aA^	92.7 ± 0.7 ^dB^
	Molded	0.4 ± 0.1 ^B^	2.1 ± 0.5 ^A^	−4.0 ± 0.7 ^A^	4.2 ± 0.6 ^B^	149.6 ± 11.1 ^A^
St-CC:LA	3D printed	0.3 ± 0.1 ^eB^	1.5 ± 0.0 ^aA^	−4.6 ± 0.5 ^dA^	5.3 ± 1.1 ^cA^	161.1 ± 1.4 ^bB^
	Molded	2.3 ± 0.2 ^A^	−0.6 ± 0.0 ^B^	−5.7 ± 0.2 ^B^	5.7 ± 0.2 ^A^	174.6 ± 1.1 ^A^
St-NE	3D printed	9.3 ± 0.4 ^bB^	−3.4 ± 0.4 ^dA^	5.9 ± 0.5 ^aB^	6.9 ± 0.5 ^bcA^	120.3 ± 4.1 ^aA^
	Molded	10.8 ± 0.6 ^A^	−3.0 ± 0.8 ^A^	4.1 ± 0.4 ^B^	4.6 ± 0.7 ^B^	114.6 ± 10.9 ^A^

Mean ± standard deviation (*n* = 3). Different ^a–d^ lowercase letters in the same column indicate significant differences in color parameters between 3D printed formulations, according to Tukey’s test (*p* < 0.05). ^A,B^ Different uppercase letters in the same column indicate significant differences in color parameters between the same 3D printed and molded formulations, according to Tukey’s test (*p* < 0.05).

**Table 6:** Parameters of texture profile analysis of 3D printed and molded gummies formulations of gelatin (Ge)- and starch (St)-based hydrogels without (C) and with NADES based on choline chloride–lactic acid (CC:LA) or NADES–annatto seed extract (NE).

Formulation	Method	Hardness (N)	Adhesiveness (-)	Springiness (mm)	Cohesiveness (mJ)	Gumminess (N)
Ge-C	3D printed	15.81 ± 2.12 ^bB^	*	1.04 ± 0.03 ^aA^	0.92 ± 0.01 ^aA^	14.48 ± 1.86 ^aB^
	Molded	24.59 ± 1.82 ^A^	18.91 ± 3.15	0.95 ± 0.01 ^B^	0.82 ± 0.04 ^B^	20.24 ± 1.05 ^A^
Ge-CC:LA	3D printed	21.04 ± 2.67 ^aB^	*	1.06 ± 0.05 ^aA^	0.88 ± 0.01 ^aA^	18.61 ± 2.38 ^aB^
	Molded	77.61 ± 2.82 ^A^	20.63 ± 2.23	0.97 ± 0.02 ^B^	0.77 ± 0.06 ^B^	60.15 ± 5.33 ^A^
Ge-NE	3D printed	20.91 ± 2.47 ^aB^	*	1.09 ± 0.04 ^aA^	0.87 ± 0.01 ^aA^	18.24 ± 2.20 ^aB^
	Molded	55.88 ± 8.91 ^A^	20.73 ± 3.91	0.95 ± 0.00 ^B^	0.69 ± 0.05 ^B^	38.59 ± 4.94 ^A^
St-C	3D printed	4.17 ± 0.37 ^cB^	1.94 ± 0.86 ^cB^	0.84 ± 0.08 ^aA^	0.48 ± 0.05 ^bA^	2.03 ± 0.28 ^bB^
	Molded	15.82 ± 2.99 ^A^	50.41 ± 2.51 ^A^	0.87 ± 0.07 ^A^	0.49 ± 0.10 ^A^	7.01 ± 0.90 ^A^
St-CC:LA	3D printed	6.76 ± 1.07 ^cB^	7.48 ± 0.92 ^aB^	0.51 ± 0.09 ^aB^	0.36 ± 0.04 ^cB^	2.46 ± 0.42 ^bB^
	Molded	17.00 ± 3.93 ^A^	28.64 ± 7.55 ^A^	0.79 ± 0.09 ^A^	0.48 ± 0.05 ^A^	7.52 ± 1.20 ^A^
St-NE	3D printed	4.51 ± 2.26 ^cB^	5.05 ± 1.20 ^bA^	0.60 ± 0.09 ^aB^	0.28 ± 0.01 ^dB^	1.29 ± 0.07 ^bB^
	Molded	10.00 ± 0.88 ^A^	2.71 ± 1.6 ^A^	0.84 ± 0.07 ^A^	0.39 ± 0.04 ^A^	3.96 ± 0.52 ^A^

* Mean ± standard deviation (*n* = 3). ^a–d^ Different lowercase letters in the same column indicate significant differences in texture profile analysis parameters between 3D printed formulations, according to Tukey’s test (*p* < 0.05). ^A,B^ Different uppercase letters in the same column indicate significant differences in texture profile analysis parameters between the same 3D printed and molded formulations, according to Tukey’s test (*p* < 0.05).

**Table 7:** Parameters of IDDSI fork test of 3D printed and molded gummies formulations in cuboids shape based on gelatin (Ge)- and starch (St)-based hydrogels without (C) and with NADES based on choline chloride–lactic acid (CC:LA) or NADES–annatto seed extract (NE), compared to the desirable parameters to reach level 5.

**Molded Samples**	**Level 5 Parameters**	**Ge-C**	**Ge-CC:LA**	**Ge-NE**	**St-C**	**St-CC:LA**	**St-NE**
Easy to mash with a fork	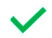	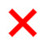	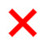	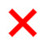	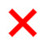	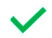	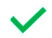
Blanched white is noted	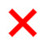	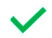	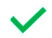	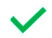	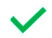	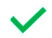	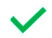
Shape recovery	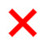	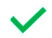	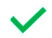	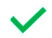	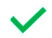	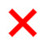	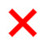
Easily broken with a fork	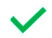	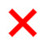	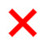	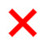	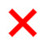	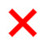	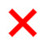
Separate thin liquid	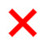	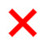	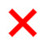	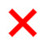	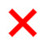	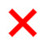	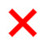
**3D printed samples**	**Level 5 parameters**	**Ge-C**	**Ge-CC:LA**	**Ge-NE**	**St-C**	**St-CC:LA**	**St-NE**
Easy to mash with a fork	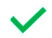	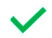	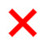	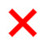	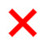	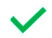	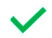
Blanched white is noted	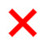	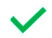	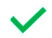	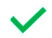	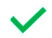	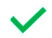	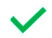
Shape recovery	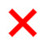	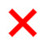	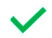	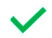	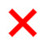	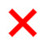	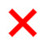
Easily broken with a fork	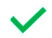	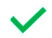	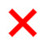	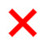	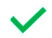	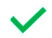	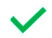
Separate thin liquid	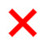	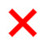	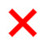	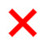	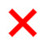	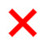	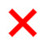

## Data Availability

The original contributions presented in this study are included in the article/Supplementary Materials. Further inquiries can be directed to the corresponding author.

## Supplementary Materials

The following supporting information can be downloaded at https://www.mdpi.com/article/10.3390/foods14091604/s1, Figure S1: Storage modulus (G′) versus temperature of scanning heat (square) and cooling (triangle), sol–gel and gel–sol transitions temperatures, and hysteresis (ΔT) of gelatin Ge-based hydrogels (Ge) without (C) and with NADES based on choline chloride–lactic acid (CC:LA) or NADES–annatto seed extract (NE).

## References

[1] Bitencourt B.S., Guedes J.S., Saliba A.S.M.C., Sartori A.G.O., Torres L.C.R., Amaral J.E.P.G., Alencar S.M., Maniglia B.C., Augusto P.E.D. (2023). Mineral Bioaccessibility in 3D Printed Gels Based on Milk/Starch/ĸ-Carrageenan for Dysphagic People. Food Res. Int..

[2] Giura L., Urtasun L., Belarra A., Ansorena D., Astiasarán I. (2021). Exploring Tools for Designing Dysphagia-Friendly Foods: A Review. Foods.

[3] Xing X., Chitrakar B., Hati S., Xie S., Li H., Li C., Liu Z., Mo H. (2022). Development of Black Fungus-Based 3D Printed Foods as Dysphagia Diet: Effect of Gums Incorporation. Food Hydrocoll..

[4] Wang Y., Zhao R., Liu W., Zhao R., Liu Q., Hu H. (2024). Effect of Twin-Screw Extrusion Pretreatment on Starch Structure, Rheological Properties and 3D Printing Accuracy of Whole Potato Flour and Its Application in Dysphagia Diets. Int. J. Biol. Macromol..

[5] Bolívar-Prados M., Baixauli R., Ismael-Mohammed K., Ortega O., Clavé P., Laguna L. (2024). Texture-Modified Foods for Patients with Swallowing and/or Mastication Impairments. A Multidisciplinary Approach to Managing Swallowing Dysfunction in Older People.

[6] Sponchiado P.A.I., de Melo M.T., Bitencourt B.S., Guedes J.S., Tapia-Blácido D.R., Augusto P.E.D., Ramos A.P., Maniglia B.C. (2024). Clean Modification of Potato Starch to Improve 3D Printing of Potential Bone Bio-Scaffolds. Emergent Mater..

[7] Montoya J., Medina J., Molina A., Gutiérrez J., Rodríguez B., Marín R. (2021). Impact of Viscoelastic and Structural Properties from Starch-Mango and Starch-Arabinoxylans Hydrocolloids in 3D Food Printing. Addit. Manuf..

[8] Wang H., Ouyang Z., Hu L., Cheng Y., Zhu J., Ma L., Zhang Y. (2022). Self-Assembly of Gelatin and Phycocyanin for Stabilizing Thixotropic Emulsions and Its Effect on 3D Printing. Food Chem..

[9] Yap K.L., Kong I., Abdul Kalam Saleena L., Pui L.P. (2022). 3D Printed Gelatin Film with *Garcinia atroviridis* Extract. J. Food Sci. Technol..

[10] Renaldi G., Junsara K., Jannu T., Sirinupong N., Samakradhamrongthai R.S. (2022). Physicochemical, Textural, and Sensory Qualities of Pectin/Gelatin Gummy Jelly Incorporated with *Garcinia Atroviridis* and Its Consumer Acceptability. Int. J. Gastron. Food Sci..

[11] Chen H., Xie F., Chen L., Zheng B. (2019). Effect of Rheological Properties of Potato, Rice and Corn Starches on Their Hot-Extrusion 3D Printing Behaviors. J. Food Eng..

[12] Kokol V., Pottathara Y.B., Mihelčič M., Perše L.S. (2021). Rheological Properties of Gelatine Hydrogels Affected by Flow- and Horizontally-Induced Cooling Rates during 3D Cryo-Printing. Colloids Surf. A Physicochem. Eng. Asp..

[13] Cheng Y., Liang K., Chen Y., Gao W., Kang X., Li T., Cui B. (2023). Effect of Molecular Structure Changes during Starch Gelatinization on Its Rheological and 3D Printing Properties. Food Hydrocoll..

[14] Maniglia B.C., Lima D.C., Matta Junior M.D., Le-Bail P., Le-Bail A., Augusto P.E.D. (2020). Preparation of Cassava Starch Hydrogels for Application in 3D Printing Using Dry Heating Treatment (DHT): A Prospective Study on the Effects of DHT and Gelatinization Conditions. Food Res. Int..

[15] de Oliveira Sartori A.G., Saliba A.S.M.C., Bitencourt B.S., Guedes J.S., Torres L.C.R., de Alencar S.M., Augusto P.E.D. (2023). Anthocyanin Bioaccessibility and Anti-Inflammatory Activity of a Grape-Based 3D Printed Food for Dysphagia. Innov. Food Sci. Emerg. Technol..

[16] Strieder M.M., Vardanega R., Moraes M.N., Silva E.K., Meireles M.A.A. (2024). One-Step Ultrasound-Assisted Recovery of Yellow-Orange-Red Natural Coloring from Defatted Annatto Seeds: A Cleaner Processing Alternative. Ultrason. Sonochem..

[17] Hirko B., Getu A. (2022). *Bixa orellana* (Annatto Bixa): A Review on Use, Structure, Extraction Methods and Analysis. J. Agron. Technol. Eng. Manag..

[18] Shridar B., Paramadhas S., Palanisamy P., Murugesan B., Kalyanasundaram K., Jayakumar J., Pandiselvam R. (2025). Development and Optimization of Temperature and Pressure-Assisted Mechanical Extraction System for Enhancing the Bixin Yield from Annatto Seeds. Biomass Convers. Biorefin..

[19] Chisté R.C., Mercadante A.Z., Gomes A., Fernandes E., da Costa Lima J.L.F., Bragagnolo N. (2011). In Vitro Scavenging Capacity of Annatto Seed Extracts against Reactive Oxygen and Nitrogen Species. Food Chem..

[20] Airouyuwa J.O., Sivapragasam N., Ali Redha A., Maqsood S. (2024). Sustainable Green Extraction of Anthocyanins and Carotenoids Using Deep Eutectic Solvents (DES): A Review of Recent Developments. Food Chem..

[21] Silveira T.M.G., Tapia-Blácido D.R. (2018). Is Isolating Starch from the Residue of Annatto Pigment Extraction Feasible?. Food Hydrocoll..

[22] Paramadhas S., Selvi P., Shridar B., Palanisamy P., Baburaj N.S., Govindarajan N., Pandiselvam R. (2024). Optimization and Extraction of Annatto Pigments Obtained from *Bixa orellana* L. Using Supercritical Fluid Extraction. Microchem. J..

[23] Jayakumar J., Sudha P., Rajkumar P., Pandiselvam R., Gurusamy K., Kumaran K., Subramanian P. (2024). Comparative Study on the Effect of Solvents on Extraction of Bixin from Annatto Seed (*Bixa orellana* L.) and Optimization of Process Parameters Using Box–Behnken Design. Biomass Convers. Biorefin..

[24] Benvenutti L., del Pilar Sanchez-Camargo A., Zielinski A.A.F., Ferreira S.R.S. (2020). NADES as Potential Solvents for Anthocyanin and Pectin Extraction from *Myrciaria cauliflora* Fruit By-Product: In Silico and Experimental Approaches for Solvent Selection. J. Mol. Liq..

[25] Bertolo M.R.V., Bogusz Junior S., Mitchell A.E. (2023). Green Strategies for Recovery of Bioactive Phenolic Compounds from Agro-Industrial Wastes (Pomegranate Peels, Almond Hulls, and Elderberry Pomace) Using Natural Deep Eutectic Solvents. ACS Food Sci. Technol..

[26] Bertolo M.R.V., Martins V.C.A., Plepis A.M.G., Bogusz S. (2021). Utilization of Pomegranate Peel Waste: Natural Deep Eutectic Solvents as a Green Strategy to Recover Valuable Phenolic Compounds. J. Clean. Prod..

[27] Fernandes C.C., Haghbakhsh R., Marques R., Paiva A., Carlyle L., Duarte A.R.C. (2021). Evaluation of Deep Eutectic Systems as an Alternative to Solvents in Painting Conservation. ACS Sustain. Chem. Eng..

[28] Jessop P.G., Jessop D.A., Fu D., Phan L. (2012). Solvatochromic Parameters for Solvents of Interest in Green Chemistry. Green Chem..

[29] Lüdtke F.L., Fernandes J., Gonçalves R.F.S., Martins J.T., Berni P., Ribeiro A.P.B., Vicente A.A., Pinheiro A.C. (2024). Performance of Β-carotene-loaded Nanostructured Lipid Carriers under Dynamic in Vitro Digestion System: Influence of the Emulsifier Type. J. Food Sci..

[30] Smith J. (2006). Annatto extracts-chemical and technical assessment. Chem Tech Assess Manual..

[31] Re R., Pellegrini N., Proteggente A., Pannala A., Yang M., Rice-Evans C. (1999). Antioxidant Activity Applying an Improved ABTS Radical Cation Decolorization Assay. Free Radic. Biol. Med..

[32] Pulido R., Bravo L., Saura-Calixto F. (2000). Antioxidant Activity of Dietary Polyphenols As Determined by a Modified Ferric Reducing/Antioxidant Power Assay. J. Agric. Food Chem..

[33] Benzie I.F.F., Strain J.J. (1996). The Ferric Reducing Ability of Plasma (FRAP) as a Measure of “Antioxidant Power”: The FRAP Assay. Anal. Biochem..

[34] Moraes I.C.F., Carvalho R.A., Bittante A.M.Q.B., Solorza-Feria J., Sobral P.J.A. (2009). Film Forming Solutions Based on Gelatin and Poly(Vinyl Alcohol) Blends: Thermal and Rheological Characterizations. J. Food Eng..

[35] IDDSI International Dysphagia Diet Standardization Initiative Framework Testing Methods 2.0. https://www.iddsi.org/images/Publications-Resources/DetailedDefnTestMethods/English/V2TestingMethodsEnglish31july2019.pdf.

[36] Fanali C., Gallo V., Della Posta S., Dugo L., Mazzeo L., Cocchi M., Piemonte V., De Gara L. (2021). Choline Chloride–Lactic Acid-Based NADES As an Extraction Medium in a Response Surface Methodology-Optimized Method for the Extraction of Phenolic Compounds from Hazelnut Skin. Molecules.

[37] Bosiljkov T., Dujmić F., Cvjetko Bubalo M., Hribar J., Vidrih R., Brnčić M., Zlatic E., Radojčić Redovniković I., Jokić S. (2017). Natural Deep Eutectic Solvents and Ultrasound-Assisted Extraction: Green Approaches for Extraction of Wine Lees Anthocyanins. Food Bioprod. Process..

[38] Jovanović M.S., Krgović N., Živković J., Stević T., Zdunić G., Bigović D., Šavikin K. (2022). Ultrasound-Assisted Natural Deep Eutectic Solvents Extraction of Bilberry Anthocyanins: Optimization, Bioactivities, and Storage Stability. Plants.

[39] Alcalde R., Gutiérrez A., Atilhan M., Aparicio S. (2019). An Experimental and Theoretical Investigation of the Physicochemical Properties on Choline Chloride–Lactic Acid Based Natural Deep Eutectic Solvent (NADES). J. Mol. Liq..

[40] Fernandes C.C., Paiva A., Haghbakhsh R., Rita C., Duarte A. (2023). Is It Possible to Correlate Various Physicochemical Properties of Natural Deep Eutectic Systems in Order to Predict Their Behaviours as Solvents?. J. Mol. Liq..

[41] Cardarelli C.R., de Toledo Benassi M., Mercadante A.Z. (2008). Characterization of Different Annatto Extracts Based on Antioxidant and Colour Properties. LWT-Food Sci. Technol..

[42] Deka B., Chakravorty P., Das A.B. (2024). Impact of Different Natural Deep Eutectic Solvents on Dissolution Behaviour and Eutectogel Structure of Jackfruit Seed Starch. J. Polym. Environ..

[43] Wilpiszewska K., Skowrońska D. (2023). Evaluation of Starch Plasticization Efficiency by Deep Eutectic Solvents Based on Choline Chloride. J. Mol. Liq..

[44] Chen G., Hu J., Liang Y., Huang X., Seah G.L., Li J., Liu D., Tan C. (2023). Protein Gel with Designed Network and Texture Regulated via Building Blocks to Study Dysphagia Diet Classifications. Food Hydrocoll..

[45] Silva-Weiss A., Bifani V., Ihl M., Sobral P.J.A., Gómez-Guillén M.C. (2013). Structural Properties of Films and Rheology of Film-Forming Solutions Based on Chitosan and Chitosan-Starch Blend Enriched with Murta Leaf Extract. Food Hydrocoll..

[46] Zhang C., Wang C.-S., Girard M., Therriault D., Heuzey M.-C. (2024). 3D Printed Protein/Polysaccharide Food Simulant for Dysphagia Diet: Impact of Cellulose Nanocrystals. Food Hydrocoll..

