# Supplementary material for: Development of NADES–Annatto Seed Extract for Enhancing 3D Printed Food Designed for Dysphagia Patients
Source: Foods. 2025 May 1;14(9):1604. doi: 10.3390/foods14091604 (PMC12072093; doi:10.3390/foods14091604)
Supplement: Supplementary file 1 [file foods-14-01604-s001.zip › foods-3593258-supplementary.pdf]

# Supplementary materials

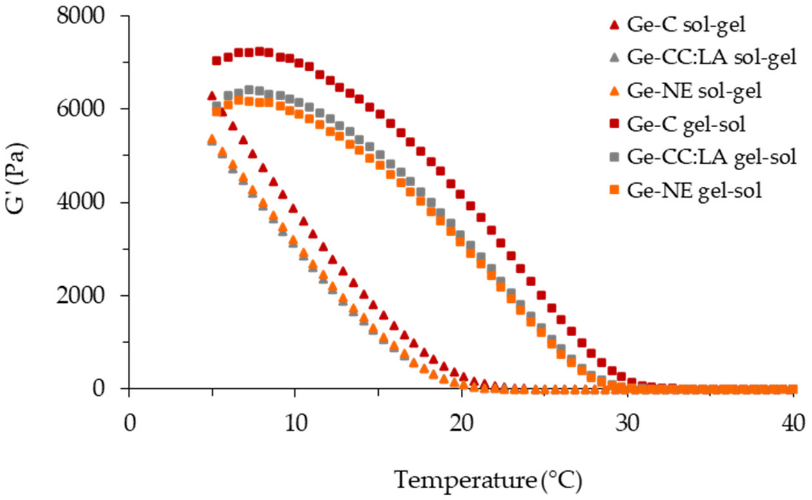


| Hydrogel | $Temperature_{sol-gel}$ (°C) | $Temperature_{gel-sol}$ (°C) | ΔT (°C) |
|---|---|---|---|
| Ge-C | $23.1 \pm 0.1^{a}$ | $32.4 \pm 0.1^{a}$ | $9.2 \pm 0.3^{a}$ |
| Ge-CC:LA | $22.2 \pm 0.6^{b}$ | $31.0 \pm 0.2^{c}$ | $8.8 \pm 0.4^{a}$ |
| Ge-NE | $22.2 \pm 0.3^{b}$ | $31.4 \pm 0.0^{b}$ | $9.2 \pm 0.3^{a}$ |

**Figure S1.** Storage modulus (G′) versus temperature of scanning heat (square) and cooling (triangle), and sol-gel and gel-sol transitions temperatures, and hysteresis (ΔT) of gelatin Ge-based hydrogels (Ge) without (C) and with NADES based on choline chloride:lactic acid (CC:LA) or NADES-annatto seed extract (NE). Means ± standard deviation (n = 3). [a-c]Different letters in the same column indicate significant differences between Ge-based hydrogels according to Tukey's test ($p < 0.05$).
